# Ranitidine Inhibition of Breast Tumor Growth Is B Cell Dependent and Associated With an Enhanced Antitumor Antibody Response

**DOI:** 10.3389/fimmu.2018.01894

**Published:** 2018-08-15

**Authors:** Dakota Rogers, Ava Vila-Leahey, Ana Clara Pessôa, Sharon Oldford, Paola A. Marignani, Jean S. Marshall

**Affiliations:** ^1^Dalhousie Inflammation Group, Department of Microbiology and Immunology, Dalhousie University, Halifax, NS, Canada; ^2^Department Biochemistry and Molecular Biology, Dalhousie University, Halifax, NS, Canada

**Keywords:** histamine receptor, breast cancer, immunology, myeloid-derived suppressor cells, natural killer cells

## Abstract

**Background:**

The histamine receptor 2 antagonist ranitidine is a commonly used, non-prescription, medication. It limits the development, growth, and metastasis of breast cancers in mouse models of disease. In this study, we examined the role of B cells in this response, the impact of ranitidine on the development of antitumor antibodies and subpopulations of natural killer cells using murine breast cancer models.

**Methods:**

Peripheral blood granulocyte populations were assessed in both E0771-GFP and 4T1 orthotopic tumor-bearing mice by evaluation of stained blood smears. Antibody responses were assessed both in terms of the levels of anti-GFP antibodies detected by enzyme-linked immunosorbent assay and also by antibody binding to the surface of tumor cells evaluated by flow cytometry. B cell and NK cell populations were examined in the draining lymph nodes and spleens of tumor-bearing animals, by flow cytometry with and without ranitidine treatment.

**Results:**

Oral ranitidine treatment was not associated with changes in peripheral blood granulocyte populations in tumor-bearing mice. However, ranitidine treatment was associated with the development of enhanced antitumor antibody responses. This was not limited to the tumor setting since ranitidine-treated mice immunized with ovalbumin also demonstrated increased IgG antibody responses. Analysis of B cell populations indicated that while B1 cell populations remained unchanged there was a significant decrease in B2 cells in the tumor-draining inguinal lymph nodes. Notably, ranitidine did not significantly inhibit primary tumor growth in B cell-deficient animals. Examination of NK cell populations revealed a significant decrease in the proportion of intermediately functionally mature NK cells populations (CD27^+^CD11b^−^) in ranitidine-treated tumor-bearing mice compared with untreated tumor-bearing controls.

**Conclusion:**

These data demonstrate an important role for B cells in the enhanced antitumor immune response that occurs in response to ranitidine treatment. Our findings are consistent with a model, whereby ranitidine reduces tumor-associated immune suppression allowing for the development of more effective antitumor responses mediated by B cells which may include the participation of NK cells. These data underline the importance of considering widely used histamine receptor antagonists as modulators of antitumor immunity to breast cancer.

## Introduction

Histamine is an important vasoactive and immune mediator, produced from various myeloid cell sources, although predominately found within mast cell and basophil granules. It is also produced by a subset of the microbiome. Histamine modulates cell activities through four distinct receptors (H1–4). It has various impacts on immune cells including antigen-presenting cells, epithelial cells, endothelial cells, natural killer cells, iNKT cells, and both T and B lymphocytes (1, 2). H1 and H4 receptors have been shown to be particularly important in the regulation of Th cell subsets and skin immune responses, respectively (3, 4), while H2 receptors are key for responses in the intestine and dendritic cell mobilization to draining lymph nodes (5, 6). Histamine has often been implicated in defective epithelial barrier function and regulation of allergic disease development but has emerged as a potent mediator of many other aspects of immune regulation over recent years (7, 8).

In the context of cancer immunology, the development and function of myeloid-derived suppressor cells has been shown to be regulated by mast cells through histamine receptors H1 and H2 (9). H2 receptors may be of particular importance in the context of breast cancer immunology since they have been shown to play key roles in regulating initial breast tumor development, tumor growth, and metastasis, through impacts on host myeloid cells (10, 11). Administration of H2 receptor antagonists in the drinking water of mice reduced primary growth in a mouse orthotopic breast cancer model, E0771; this process was dependent on CCL2 and could be inhibited by low dose gemcitabine treatment, consistent with an MDSC-dependent mechanism of action (11). In mice that were genetically susceptible to spontaneous breast cancer development treatment with ranitidine in the drinking water from the time of weaning reduced the number of breast tumors developed in the mice by 50% compared with untreated mice (10). Natural killer cells are also known to be important for immune surveillance and effective anticancer immunity. Histamine treatment in combination with IL-2 therapy has been shown to lead to the development of altered NK cell subpopulations (12). NK cells are known to express H4 receptors and H2 receptors (13, 14). NK cell targets might also be modulated by the presence of histamine altering expression of NKG2D (15). The activity of NK cells in tumor settings can be enhanced through the presence of antitumor antibodies. The inhibitory actions of MDSC on CD4 T cells might be expected to limit or modify the nature of such antibody responses in tumor-bearing mice. Previous studies have shown that targeting H2 signaling can alter antibody secretion by B cells (16, 17). These studies focus on T cell-dependent antibody production and suggest a key role for histamine in regulating T cell function, and therefore indirectly altering antibody production.

Previous studies have suggested that lack of H1 function is associated with increased antibody responses to OVA immunization, while a deficiency in H2 receptors had little impact on such responses in mice (16, 18). However, given the key role of histamine receptors in T cell activation and CD4 T cell polarization, such modulation of antibody production could occur due to impacts on either T cells or B cells. Furthermore, other histamine receptor expressing cells such as monocytes, neutrophils, and dendritic cells can also influence both T and B cell responses. Direct evaluation of the impact of H2 receptor blockade on antibody responses to breast tumors has not been reported, to our knowledge.

Clinically, H2 receptor antagonists are widely used for the treatment of gastrointestinal disorders. In this study, we have further pursued the mechanism of ranitidine tumor growth inhibition through examination of the role of B cells, the development of antibody responses, and modulation of NK cell populations in the context of both control and breast tumor-bearing animals.

## Materials and Methods

### Cell Lines

Mouse epithelial breast carcinoma cell line 4T1 and mouse breast adenocarcinoma cell line E0771 (NCI) transduced with turboGFP (Evrogen, Moscow, Russia) and mouse melanoma cell line B16.F10 (ATCC) transduced with OVA were maintained in a monolayer in Dulbecco’s modified Eagle’s medium (Hyclone) containing 10% fetal bovine serum, and 1% l-glutamine, HEPES, and penicillin/streptomycin. Selection for GFP-positive E0771 cells were maintained with 4 µg/mL of puromycin in the media. OVA-positive B16.F10 cells were maintained with 500 µg/mL G418 in the media.

### Mice

All mouse experiments were pre-approved by the Dalhousie University Committee on Laboratory Animals. Five-week-old female BALB/c mice and C57BL/6 mice were purchased from Charles River Laboratories. Five-week-old female B6.129S2-*Ighm^tm1Cgn^*/J mice (muMt^−/−^ B cell-deficient mice) on a C57BL/6 genetic background were purchased from the Jackson Laboratory. B6.129P2-*Fcgr3^tm1Sjv^*/SjvJ mice (FcγRIII^−/−^ mice) and B6.129S4-*Ccr2^tm1lfc^*/J (CCR2^−/−^ mice) on a C57BL/6 genetic background were bred in house. Lkb1^−/−^/NIC were as previously described (19). All mice were housed in specific pathogen-free conditions at the Carleton Animal Care Facility at Dalhousie University.

### *In Vivo* Cancer Models

Histamine antagonists were added to drinking water 1 day prior to tumor cell injection and were refreshed every other day. An adapted protocol was employed for orthotopic models (20). For the E0771-GFP model, 6- to 8-week-old female C57BL/6 mice were anesthetized and 200,000 cells in 100 µL of Matrigel^®^ (Corning) were injected subcutaneously into the mammary fat pad near the fourth nipple. The volume of the tumor was determined by caliper measurements every second day using the equation volume = length × width^2^/2. For the 4T1 model, 6- to 8-week-old BALB/c female mice were anesthetized and 100,000 4T1 cells in 50 µL PBS were injected subcutaneously into the mammary fat pad near the fourth nipple. For the B16-OVA model, 6- to 8-week-old female mice were anesthetized, and 100,000 B16-OVA cells in 50 µL PBS were injected subcutaneously into the back flank. The volumes of the tumors were measured as previously stated above. At day 19 post injection for the E0771-GFP and 4T1 models, and day 21 post injection for the B16-OVA model, the mice were sacrificed, and the primary tumor, spleen, and tumor-draining inguinal lymph node were collected.

### Blood Smear and Staining

On the day of tumor cell implant, and days 7, 14, and 19 after implant, 4T1 and E0771 tumor-bearing mice were restrained and 100 µL of blood was isolated by puncturing the submandibular vein with a lancet. Circulating leukocyte concentrations were counted on a hemocytometer using 3% acetic acid in methylene blue. Approximately 10 µL of blood was used for a blood smear on microscope slides and then allowed to dry overnight. A modified protocol of blood staining was performed using Differential Quik Stain Kit (Electron Microscopy Sciences). The samples were then mounted with DPX mounting medium (Sigma-Aldrich) and viewed under a light microscope at 400×.

### Flow Cytometric Assessment of Tumor-Specific Antibodies

Secondary antibodies: rat anti-mouse IgG2a-bio (BioLegend), rat anti-mouse Igλ-bio (BD Biosciences), rat anti-mouse Igκ-bio (BD Biosciences), and rat anti-mouse Igκ-FITC (BD Biosciences). Streptavidin (SA)-conjugated detection proteins: PE-SA (eBioscience) and APC-SA (BioLegend).

E0771-GFP cells or SK-BR-3 cells (a Her2-positive cell line) were routinely cultured. Cells were then blocked in FACS buffer containing human IgG (1 μL/50 μL FACS buffer). Mouse serum, obtained from tumor-bearing or control animals, was added to the cells at dilutions of 1/10 and 1/100, and the cells were incubated on ice for 15 min. Cells were washed and biotinylated secondary anti-mouse-Ig antibodies were added and incubated for 15 min on ice. Again, cells were washed, and SA-conjugated fluorochromes were added, and the cells were fixed with 1% paraformaldehyde. Stained cells were acquired for analysis using a BD FACSCalibur, and results were analyzed using FCS express software. The amount of antibody binding to whole E0771-GFP tumor cells was quantified by relative fluorescence intensity (relative to the average mean fluorescence intensity of control group).

### Flow Cytometric Analysis of Lymphocytes

Primary antibodies: rat anti-mouse CD11b-fluorescein isothiocyanate (FITC) (eBioscience), rat anti-mouse CD45-APC (BioLegend), rat anti-mouse CD45-biotin (bio) (BioLegend), rat anti-mouse CD11b-PE (eBioscience), rat anti-mouse Ly6C-APC (BioLegend), rat anti-mouse Ly6G-bio (BioLegend), rat anti-mouse CD49d-PE (eBioscience), rat anti-mouse CD11b-PECy7 (eBioscience), mouse anti-mouse NK1.1-bio (eBioscience), Armenian hamster anti-CD27-PE-Cy7 (eBioscience), rat anti-mouse NKG2D-PE (eBioscience), rat anti-mouse CD3-APC (eBioscience), rat anti-mouse CD16/CD32-PE-Cy7 (eBioscience), rat anti-mouse CD3-PE (BioLegend), rat anti-mouse CD127-APC (eBioscience), rat anti-mouse CD19-FITC (eBioscience), rat anti-mouse CD21/CD35-PerCP-eflour710 (eBioscience), rat anti-mouse CD23-bio (eBioscience), rat anti-mouse CD43-PE (eBioscience), rat anti-mouse MHCII-Alexa Fluor 700 (BioLegend), and rat anti-mouse CD138-APC (BioLegend).

Streptavidin-conjugated detection proteins: PerCP-SA (BioLegend), APC-eFluor780-SA (eBioscience), and APC-SA (BioLegend).

Isotype control antibodies: rat IgG2a-Alexa Fluor 647 (BioLegend), rat IgG2a-bio (eBioscience), rat IgG2b-FITC (eBioscience), rat IgG2b-PE (BioLegend), mouse IgG2a-bio (eBioscience), Golden Syrian Hamster IgG-PECy7 (eBioscience), rat IgG1-PE (BioLegend), rat IgG2b-APC (BioLegend), and rat IgG2a-PECy7 (eBioscience).

Splenocytes and tumor-draining inguinal lymph nodes from control and ranitidine-treated tumor-bearing mice were processed into single-cell suspensions. After the addition of the primary antibodies, the samples were then washed with FACS buffer and SA-conjugated proteins were added and incubated at 4°C for 20 min. Stained cells were acquired for analysis using a BD LSRFortessa, and results were analyzed using FCS express software.

### Enzyme-Linked Immunosorbent Assay (ELISA)

Enzyme-linked immunosorbent assay plates were coated with anti-mouse IgG1 (BioLegend) or anti-mouse IgG2a (BioLegend) at a concentration of 2.5 µg/mL in borate buffer (2.5 µg/mL, pH 8.3) and placed at 4°C overnight. The plates were then washed and blocking buffer [2% fish gelatin (Sigma-Aldrich) or 2% BSA (Roche, Laval, QC, Canada) diluted in PBS] was added and incubated at room temperature for 2 h. Plates were then washed, and diluted serum from E0771-GFP or B16-OVA tumor-bearing mice or OVA-immunized mice were added and incubated at 4°C overnight. Serum from a naïve non-tumor-bearing or non-immunized C57BL/6 mouse was used as a negative control. For the anti-OVA antibody detection, pooled sera from OVA-immunized mice were used as a positive control. For anti-GFP analysis, experiments concurrently analyzed groups of mice sera were compared. Horse radish peroxidase and SA antibody detection systems were employed according to the manufacturer’s instructions.

### OVA-Alum Immunization

Female C57BL/6 mice (8–10 weeks old) were immunized with OVA precipitated with aluminum potassium sulfate (alum) (Sigma-Aldrich). The mice were divided into control and treatment (ranitidine in drinking water, 8 mg/kg/mouse/day) groups, 1 day prior to immunization and refreshed as stated above. 50 µL of 1 mg/mL OVA-alum was diluted into 50 µL of sterile PBS. This 100 µL mixture was then injected intraperitoneally (i.p.) into each mouse. 14 days post-immunization, the mice were boosted with 10 µL of 1 mg/mL OVA, without adjuvant, diluted in 90 µL of sterile PBS. On day 21, the mice were sacrificed and blood was collected for detection of antibodies against OVA by ELISA.

### Statistical Analysis

All flow cytometry, ELISA, and tumor weight comparison data were analyzed with one-way ANOVA, two-way ANOVA, or Student’s unpaired *t*-tests using Prism software, as appropriate. *Post hoc* analyses were performed using Fisher’s exact test as indicated. Correlation analyses for flow cytometry, ELISA, and tumor weight were performed using Prism software.

## Results

### Analysis of the Impact of Ranitidine on Myeloid Cells in the Peripheral Blood of Tumor-Bearing Mice

Previous work in our laboratory has shown that ranitidine treatment caused a decrease in breast tumor growth and metastasis (10) and also decreased the immunosuppressive activity of peripheral blood cells. To further analyze the impact of ranitidine on immune effector cells blood samples from 4T1 and E0771-GFP tumor-bearing mice were examined for the major Fc receptor-bearing populations (neutrophils, monocytes, and eosinophils). In the 4T1 model, there was an increase in the percent of blood neutrophils over the course of tumor growth, with a subsequent decrease in the percent of monocytes (Table 1). There were no alterations in the proportion of eosinophils in the circulation. In the E0771-GFP model, there was a similar increase in neutrophils over the course of tumor growth and no alteration in eosinophils. However, there was no decrease in monocytes over time in this model (Table 2). Ranitidine treatment had no significant impact on the overall numbers of these peripheral blood cell populations in tumor-bearing mice.

**Table 1:** Percent of neutrophils, monocytes, and eosinophils of live leukocytes in circulation in 4T1 tumor-bearing BALB/c mice.

Day	% Neutrophils		% Monocytes		% Eosinophils	

	Control	Ranitidine	Control	Ranitidine	Control	Ranitidine
0	15.13 ± 1.46	9.13 ± 1.68*	19.50 ± 2.96	12.94 ± 3.06 (NS)	2.00 ± 0.29	2.25 ± 0.79 (NS)
7	18.00 ± 2.51	18.63 ± 2.14 (NS)	7.38 ± 0.80	6.69 ± 1.39 (NS)	1.13 ± 0.43	1.63 ± 0.55 (NS)
14	46.00 ± 14.40	43.36 ± 5.31 (NS)	4.50 ± 1.51	4.29 ± 0.49 (NS)	1.38 ± 0.66	2.07 ± 0.39 (NS)
19	58.25 ± 2.52	54.21 ± 4.01 (NS)	0.42 ± 0.36	0.03 ± 0.01 (NS)	1.50 ± 0.54	0.94 ± 0.24 (NS)

NS, non-significant, *p < 0.05, Student’s unpaired t-test, N = 4–8.

**Table 2:** Percent of neutrophils, monocytes, and eosinophils of live leukocytes in circulation in E0771-GFP tumor-bearing C57BL/6 mice.

Day	% Neutrophils		% Monocytes		% Eosinophils	

	Control	Ranitidine	Control	Ranitidine	Control	Ranitidine
0	9.75 ± 2.33	15.13 ± 2.01 (NS)	1.50 ± 0.54	4.13 ± 0.43**	0.88 ± 0.88	1.75 ± 0.43 (NS)
7	14.88 ± 3.22	15.13 ± 2.88 (NS)	4.75 ± 0.52	4.75 ± 0.60 (NS)	2.50 ± 0.54	2.63 ± 0.90 (NS)
14	13.13 ± 2.83	11.13 ± 4.32 (NS)	6.50 ± 1.14	5.50 ± 1.14 (NS)	2.25 ± 0.66	2.63 ± 0.59 (NS)
18	25.50 ± 4.26	24.38 ± 5.72 (NS)	4.25 ± 0.75	5.88 ± 0.80 (NS)	1.38 ± 0.43	2.38 ± 0.66 (NS)

NS, non-significant, **p < 0.01, Student’s unpaired t-test, N = 4.

### Ranitidine-Treated Mice Have Increased Levels of Antitumor Antibodies

**Figure 1:**
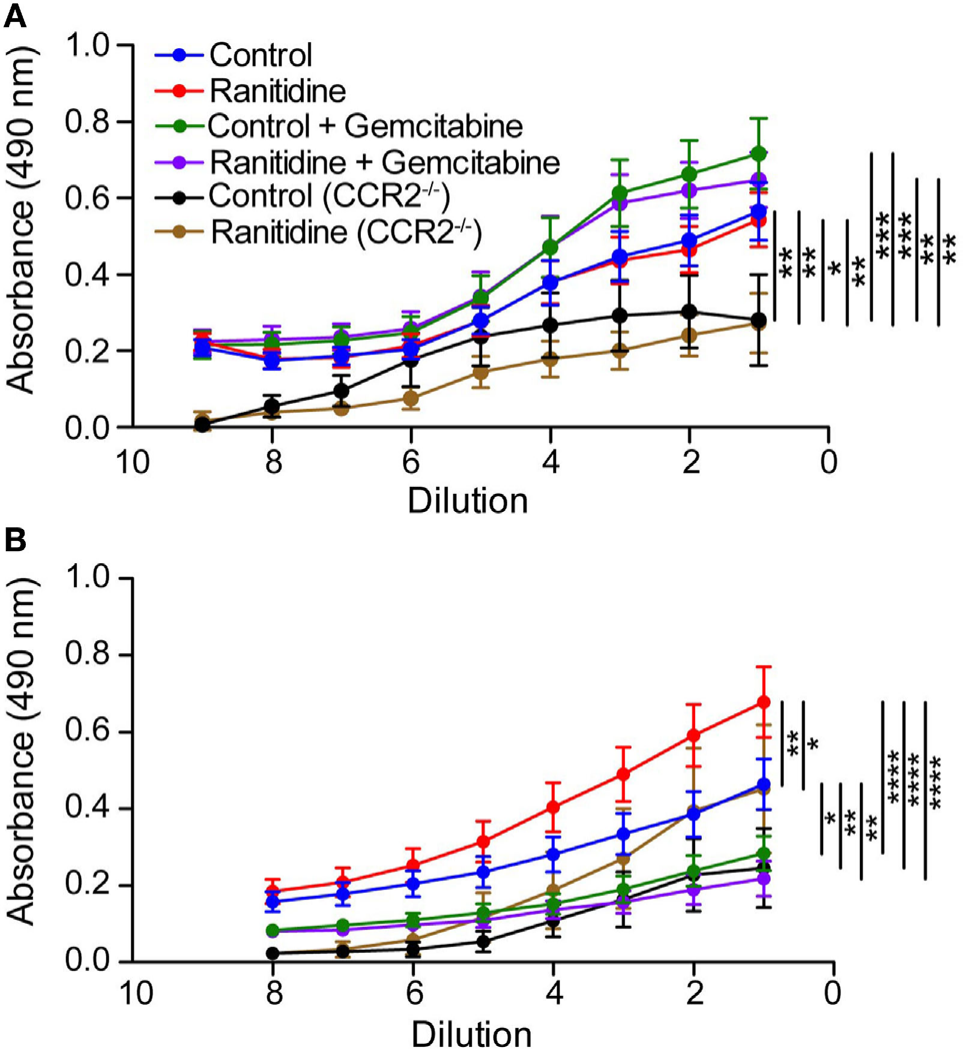
Alterations in anti-GFP IgG1 and IgG2a antibody in ranitidine-treated E0771-GFP tumor-bearing mice. Plasma from C57BL/6 and CCR2^−/−^ tumor-bearing mice (treated and non-treated) were serially diluted and an enzyme-linked immunosorbent assay was used to detect GFP-specific IgG1 **(A)** or IgG2a **(B)**. Data points represent mean ± SEM of 10–34 mice. Significance was assessed using two-way ANOVA followed by uncorrected Fisher’s LSD multiple comparisons. *p < 0.01, **p < 0.01, ***p < 0.001, and ****p < 0.0001.

Ranitidine inhibits E0771-GFP tumor growth when administered orally, as we have previously reported (10, 11). The IgG1 and IgG2a antibody responses against tumor-associated GFP were analyzed. No differences were observed between ranitidine-treated and non-treated mice in terms of GFP-specific IgG1 antibody (Figure 1A), but there was evidence of increased levels of GFP-specific IgG2a in ranitidine-treated mice as opposed to non-treated tumor-bearing mice (Figure 1B). When mice were treated with gemcitabine, a known depressor of B cell function and antibody production (11), IgG1 antibody production was unaffected, whereas IgG2a antibody production was almost completed ablated in both control and ranitidine-treated tumor-bearing mice (Figure 1). In samples from E0771-GFP tumor-bearing CCR2^−/−^ mice where ranitidine has previously been shown to not inhibit tumor growth (11), overall, IgG1 and IgG2a antibody production was decreased compared with wild-type C57BL/6 mice and ranitidine treatment was associated with an increase in GFP-specific IgG2a, albeit not significant (Figure 1).

**Figure 2:**
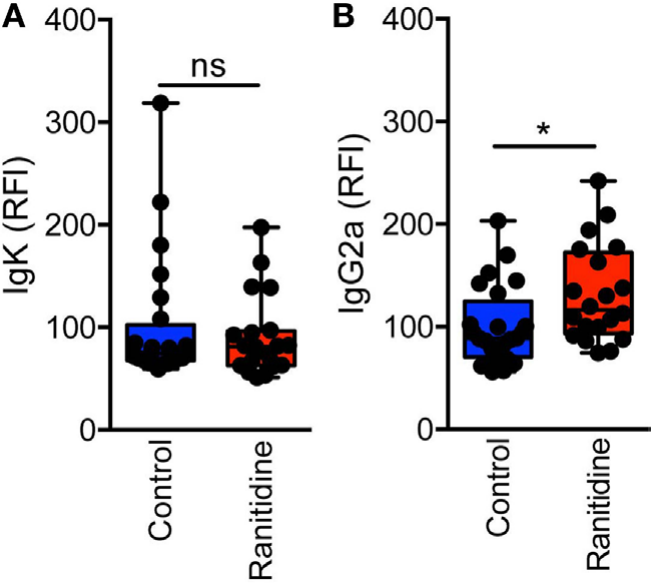
Ranitidine significantly enhanced levels of IgG2a capable of binding to E0771-GFP cancer cells. Serum was diluted 1:10 and added to cultured E0771-GFP cells and anti-isotype antibodies for IgG2a **(A)** or kappa light chain **(B)** were added. Relative fluorescence intensity (RFI) was used as an indicator for antibody binding to E0771-GFP cells relative to the average of control MFI. Box plot represents mean and range of data from 20 to 24 individual mice. *p < 0.05, unpaired *t*-test.

**Figure 3:**
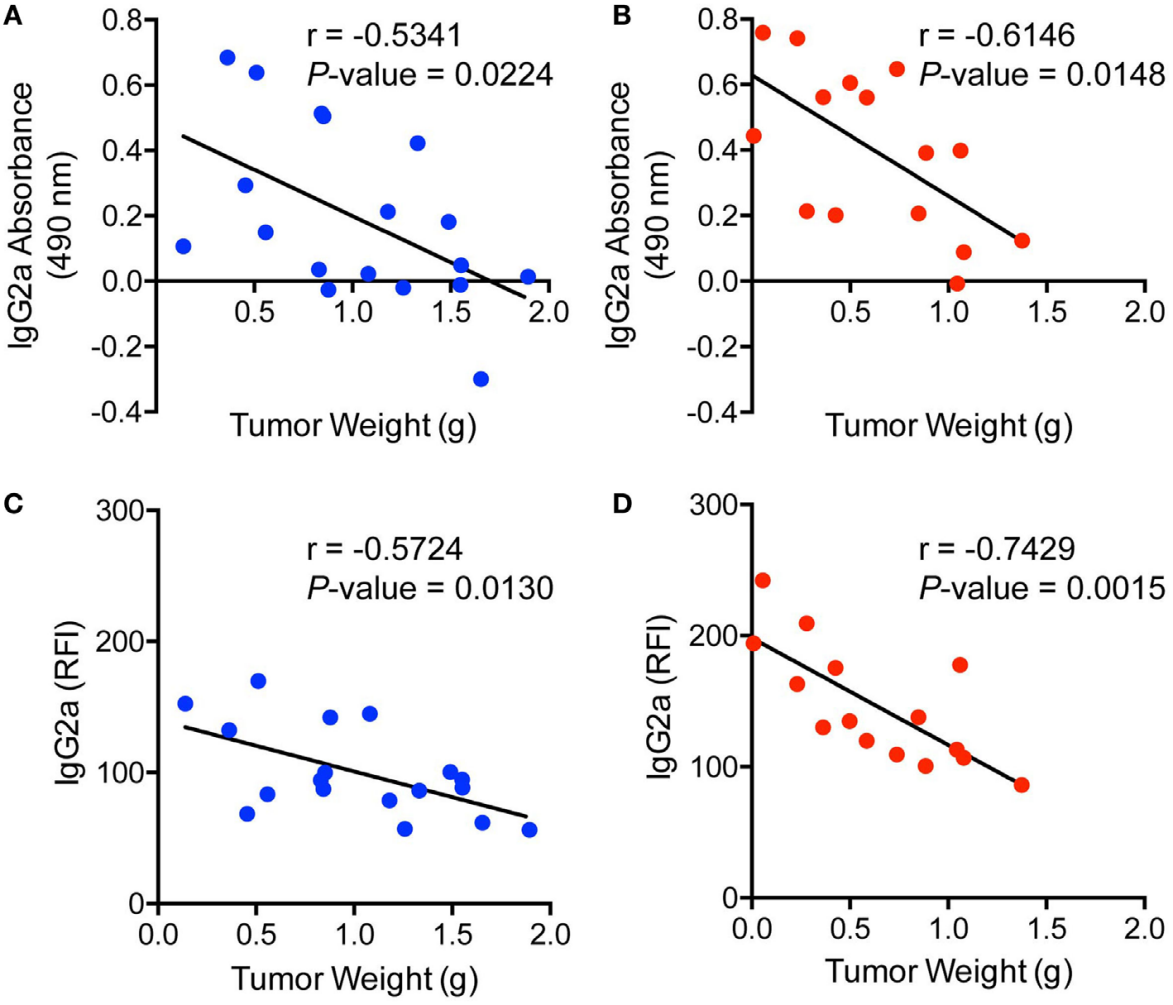
Ranitidine enhances increased IgG2a antibody production and binding which significantly correlates with tumor weight. Tumor weights from non-treated **(A,C)** or ranitidine-treated mice **(B,D)** were plotted relative to the amount of IgG2a antibody that is specific to GFP **(A,B)** or to E0771-GFP tumor cell-binding IgG2a **(C,D)**. Each dot represents individual mice and lines represent the correlation line. Correlation analyses were performed to obtain *r* and *p*-value.

To further examine the levels of antibody binding to E0771-GFP cells in both ranitidine-treated and control tumor-bearing mice, plasma from tumor-bearing mice was added to E0771-GFP cells, followed by anti-IgG2a or anti-kappa chain detection antibodies, and analyzed by flow cytometry. There was no difference in total antibody binding to E0771-GFP tumor cells from plasma derived from ranitidine-treated tumor-bearing mice (Figure 2A); however, when explored further, ranitidine treatment was associated with significantly increased IgG2a antibody directed against E0771-GFP tumor cells (Figure 2B). When comparing the antibody levels with tumor size, it was shown that tumor size was inversely correlated with the amount of tumor antigen-specific antibody and tumor cell-specific antibody present, in both non-treated and ranitidine-treated mice, with a strong correlation in ranitidine-treated mice (Figure 3).

In a separate series of experiments, SK-BR-3 cells (a Her2-positive cell line) were used to determine whether there was anti-Her2 antibody produced in the Lkb1^−/−^/NIC mice that were used as a model of spontaneous breast tumor development (19) in which we had previously demonstrated a reduced level of tumor development in the context of ranitidine treatment (10). We employed an anti-Her2 IgG antibody as a positive control for the stain. Utilizing the same protocol we performed to determine E0771-GFP-specific antibodies, we found a significant increase in IgG1 antibody production against SK-BR-3 cells with ranitidine treatment in mice that had not yet developed tumors when compared with similar control treated mice (Figure S1 in Supplementary Material).

### Ranitidine-Treated Mice Show Enhanced Antibody Responses to OVA Immunization

**Figure 4:**
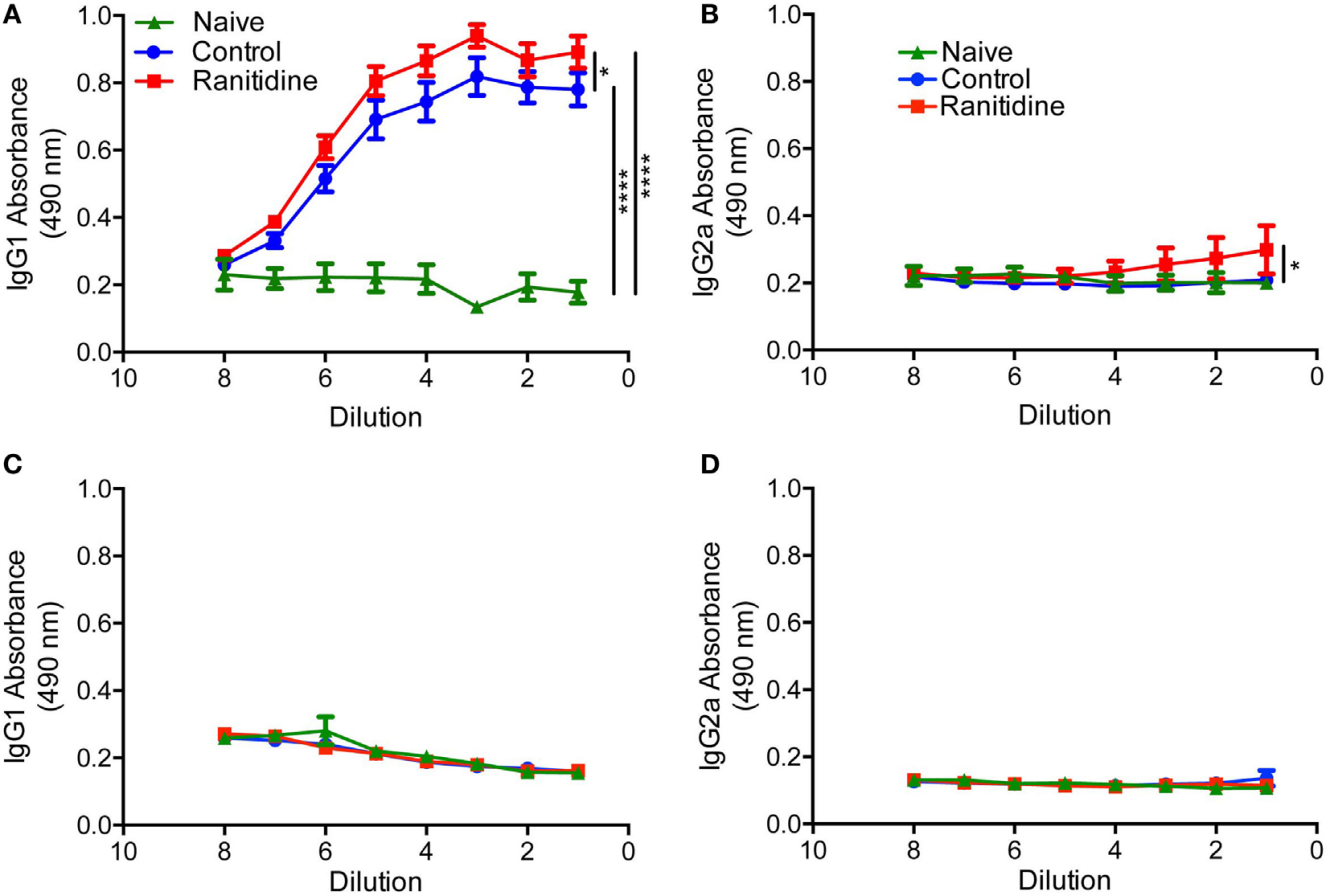
Ranitidine enhanced anti-OVA IgG2a and IgG1 antibody levels in OVA-Alum immunized naïve C57BL/6 mice but not in B16-OVA tumor-bearing mice. C57BL/6 mice were immunized and boosted with OVA with alum as an adjuvant **(A,B)**. C57BL/6 mice (ranitidine-treated or non-treated) were implanted with OVA-expressing B16 tumor cells **(C,D)**. After 18 days, the plasma from the mice were serially diluted and enzyme-linked immunosorbent assay was used to detect OVA-specific IgG1 **(A,C)** and IgG2a **(B,D**). Data points represent mean ± SEM of 12 mice. Significance was assessed using two-way ANOVA followed by uncorrected Fisher’s LSD multiple comparisons. *p < 0.05 and ****p < 0.0001.

To determine whether the effect of ranitidine on antibody responses was tumor specific, we utilized an OVA-Alum immunization and boost protocol to determine whether ranitidine altered OVA-specific antibody responses. ELISA results revealed that ranitidine-treated mice had higher titers of OVA-specific IgG1 and IgG2a compared with mice that were immunized without ranitidine treatment (Figures 4A,B). However, when the B16.F10-OVA melanoma model was used, there was no OVA-specific IgG1 and IgG2a antibody production observed (Figures 4C,D). This suggests that while ranitidine is able to increase antibody production in an OVA immunization model and in the E0771 breast cancer model it does not have this effect in all tumor models, where there may be other immunosuppressive elements that ranitidine does not alter (11).

### The Tumor Growth Limiting Effect of Ranitidine Is B Cell-Dependent

**Figure 5:**
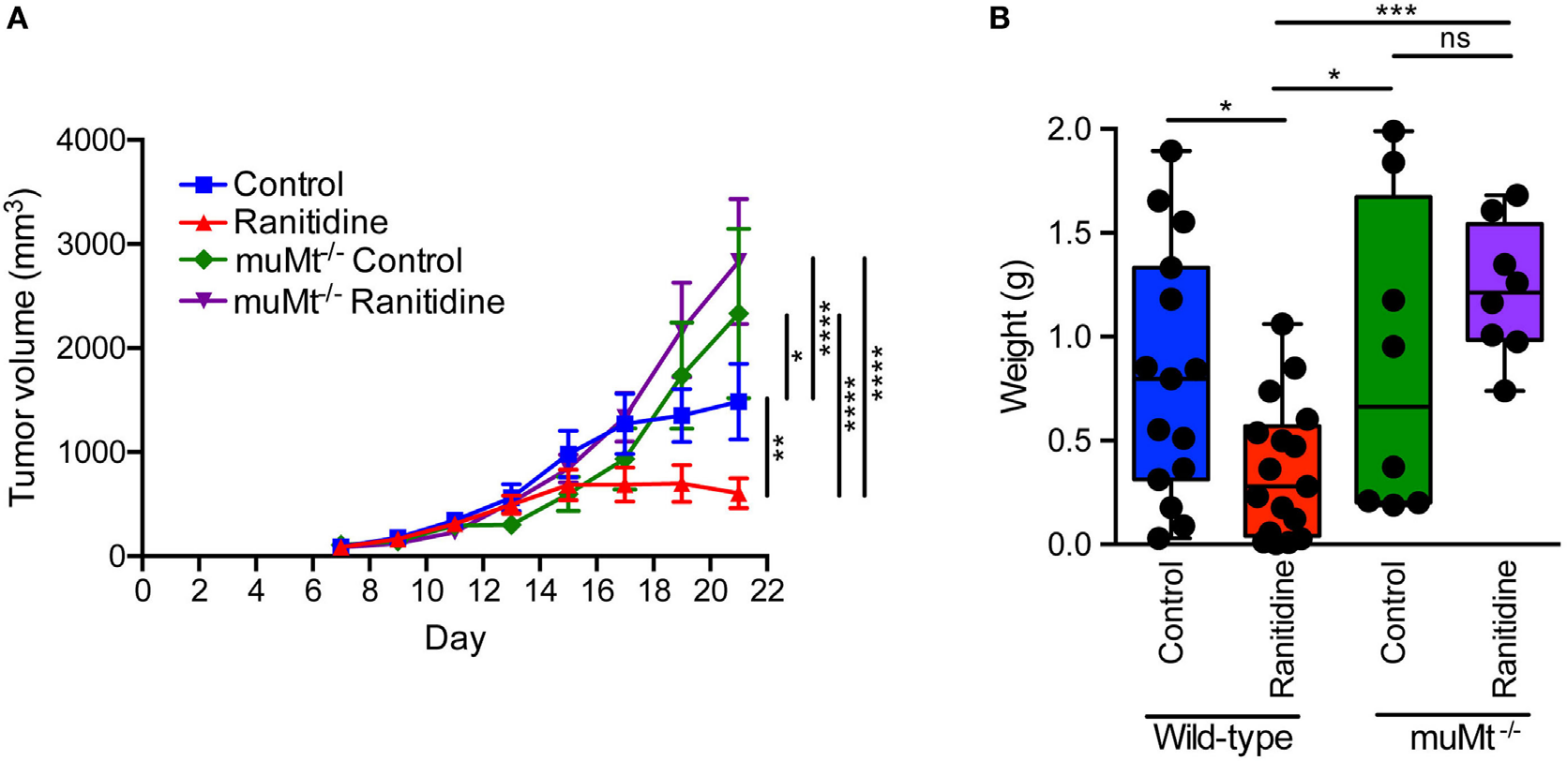
Ranitidine does not impact E0771-GFP tumor growth in B cell-deficient C57BL/6 mice. **(A,B)** E0771-GFP tumors in muMt^−/−^ and wild-type C57BL/6 mice treated with ranitidine (8 mg/kg) were measured every 2 days starting 7 days post E0771-GFP cell injection. **(A)** At day 21, the primary tumor was excised and weighed **(B)**. Data in panel **(A)** represents the mean ± SEM tumor volume of 8–17 mice/point, significance was assessed using two-way ANOVA with uncorrected Fisher’s LSD multiple comparisons. Data points in panel **(B)** represent final tumor weight of individual mice, significance was assessed using two-way ANOVA with uncorrected Fisher’s LSD multiple comparisons. *p < 0.05, **p < 0.01, ***p < 0.001, ****p < 0.0001, NS, not significant.

B cell-deficient C57BL/6 mice (muMt^−/−^) were utilized to determine whether the impact of ranitidine treatment on E0771-GFP tumor growth was dependent on the presence of B cells. The results showed that compared to wild-type C57BL/6 mice, E0771-GFP tumor growth was greater in muMt^−/−^ mice, suggesting that B cells are important in inhibiting tumor growth in this model. With ranitidine treatment, there was no significant impact on E0771-GFP tumor growth in muMt^−/−^ mice. By contrast, in wild-type mice, the final tumor weights were significantly decreased with ranitidine treatment when compared with non-treated animals, in muMt^−/−^ mice, there was no significant difference in final tumor weight between ranitidine-treated and non-treated mice (Figure 5). Since B cells express H2 receptors (21) it is possible that one mechanism of ranitidine-dependent tumor growth inhibition is by directly regulating B cell function and enhancing antibody production targeted at the tumor cells.

### B Cell Populations in Tumor-Bearing Mice With Ranitidine Treatment

**Figure 6:**
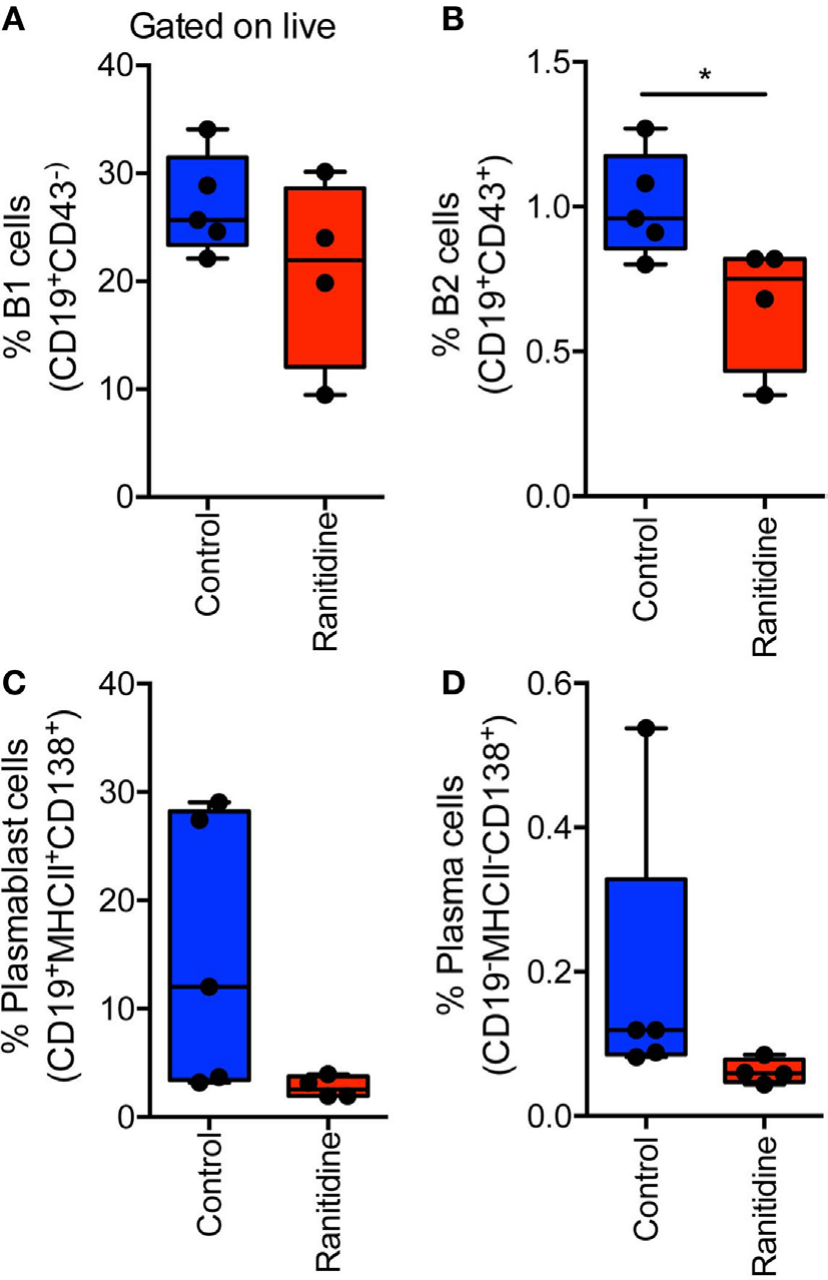
Ranitidine significantly decreases B2 cells in the draining lymph nodes of E0771-GFP tumor-bearing C57BL/6 mice. Draining inguinal lymph nodes were isolated from E0771-GFP tumor-bearing mice (ranitidine-treated and control) and cells were stained for B1 cells [CD19^+^CD43^−^; **(A)**], B2 cells [CD19^+^CD43^+^; **(B)**], plasmablasts [CD19^+^MHCII^+^CD138^+^; **(C)**], and plasma cells [CD19^−^MHCII^−^CD138^+^; **(D)**]. Boxplots represent mean and range from individual mice. *p < 0.05, unpaired *t*-test.

E0771-GFP tumor-bearing C57BL/6 mice spleens were isolated to analyze B cell populations, specifically, B2 cells (CD19^+^CD43^+^) and B1 cells (CD19^+^CD43^−^). Within the B1 cell population, cells were also separated into Marginal Zone B cells (CD23^int^CD21^+^) and Follicular B cells (CD23^+^CD21^−^). Flow cytometry results showed that there were no significant alterations in any of these splenic B cell populations with ranitidine treatment (Table S1 in Supplementary Material). However, analysis of B cells in the draining inguinal lymph node revealed that there was a significant decrease in B2 cells (Figure 6) in this location in ranitidine-treated animals when compared with non-treated controls.

### NK Cell Populations in Tumor-Bearing Mice With Ranitidine Treatment

**Figure 7:**
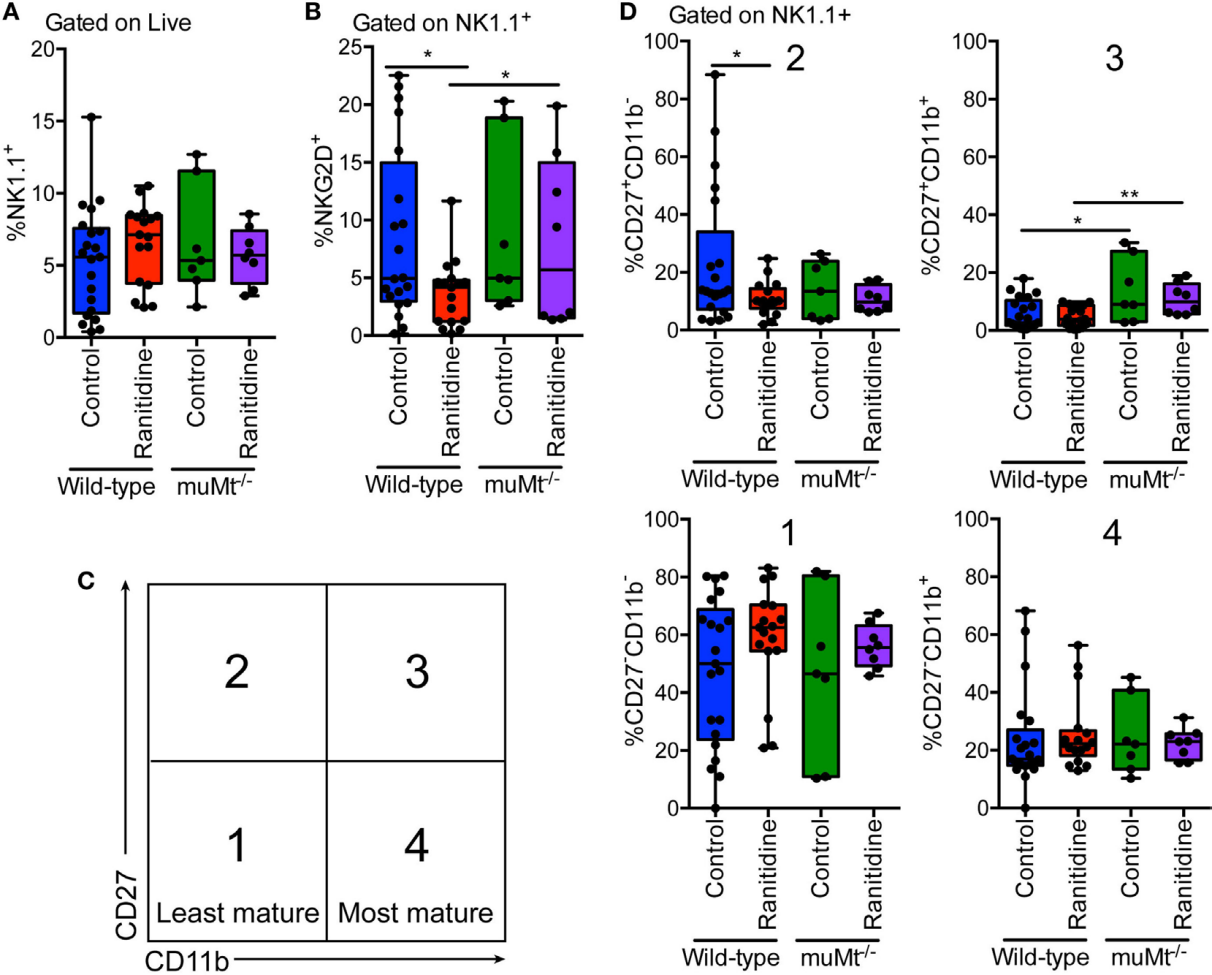
NK cell maturation and NKG2D expression was significantly altered in wild-type E0771-GFP tumor-bearing mice with ranitidine treatment, but not in muMt^−/−^ B cell-deficient mice. Spleens were isolated from tumor-bearing wild-type and B cell-deficient C57BL/6 mice (ranitidine-treated and control) and stained for **(A)** total NK cells (NK1.1^+^), **(B)** activated NK cells (NK1.1^+^NKG2D^+^), and **(C,D)** NK cell maturation markers CD27 and CD11b. Boxplots represent mean and range from individual mice. *p < 0.05, **p < 0.01, unpaired *t*-test.

NK cells are known to express H2 receptors and have been reported to be influenced by histamine receptor blockade (12–15). Given the importance of NK cells in antitumor immunity and the role of antibodies in enhancing their killing activity we examined splenic NK cell populations. In both wild-type and muMt^−/−^ tumor-bearing mice, ranitidine did not affect the total NK cell population (Figure 7A). However, impacts of ranitidine treatment were observed upon further analysis of NK cell functional maturation markers (CD27 and CD11b) and NKG2D expression. Flow cytometry analysis showed that with ranitidine treatment, there was a significant decrease in intermediately functionally mature NK cells, CD27^+^CD11b^−^ (Figures 7C,D). Analysis of the same populations in muMt^−/−^ tumor-bearing mice showed a significant increase in the late intermediately mature splenic NK cell population, CD27^+^CD11b^+^, between the control wild-type and muMt^−/−^, and the ranitidine-treated wild-type and muMt^−/−^ mice (Figures 7C,D). Furthermore, in wild-type mice, there was a significant decrease in NKG2D^+^ NK cells following ranitidine treatment. Notably, in muMt^−/−^ mice, the percentage of NKG2D expressing NK cells was significantly increased in ranitidine-treated muMt^−/−^ tumor-bearing mice compared to wild-type ranitidine-treated mice (Figure 7B).

## Discussion

Our previous studies have demonstrated that oral ranitidine treatment can reduce solid tumor growth in the E0771-GFP model, inhibit tumor metastasis in the 4T1 model, and limit the development of tumors in a targeted Lkb1-deficient breast cancer model (10). These changes correlated with a reduction in MDSC populations following ranitidine treatment. MDSCs have been implicated in reducing effective antitumor immune responses at both cellular and humoral levels [reviewed in Ref. (22)]. This study evaluated changes in antibody responses and relevant populations of Fc receptor-bearing cells in tumor-bearing animals with and without concurrent ranitidine treatment. We hypothesized that given the evidence of reduced tumor-associated immune suppression in the presence of ranitidine we would observe a greater antibody response. This could potentially contribute to effective antitumor host defense through antibody-dependent cellular cytotoxicity, complement fixation and other mechanisms. Our data indicate that ranitidine treatment significantly increased the antibody response to tumor-associated antigens, including both the intracellular GFP expressed by our tumor model and extracellular tumor antigens assessed by flow cytometry. These changes occurred in the absence of any significant impact of the ranitidine treatment on neutrophil or eosinophil numbers within the peripheral blood.

The development of antibody responses against tumors has been studied in multiple contexts. In recent studies of effective immunotherapy, the development of antibodies to tumor-associated antigens has been associated with a positive response (23, 24). However, the importance of antibodies as a component of effective antitumor responses has long been recognized (25). In this study, ranitidine is thought to function at least in part through modulation of tumor-associated immune suppression and would be predicted to impact multiple arms of acquired immune function, including antibody development. Both the IgG2a response to GFP, which is intracellular in location, and to cell surface tumor antigens was enhanced in the context of ranitidine treatment. An enhanced IgG1 response to a Her2 positive tumor cell line was also observed using the spontaneous Lkb1^−/−^/NIC model, demonstrating that the impact of ranitidine on antibody responses was not restricted to the E0771 model. The ability of oral ranitidine treatment to also enhance the antibody response to ovalbumin confirms that the positive impact of ranitidine treatment on antibody responses is not restricted to a tumor setting, suggesting that its actions in enhancing humoral immunity are not limited to alleviating tumor-associated immune suppression.

Jutel et al. (16, 26) have reported that both Th1 and Th2 responses are negatively regulated by H2 through the activation of distinct mechanisms and defined key roles for this receptor in immune regulation. This is in keeping with our findings of enhanced antibody responses to both tumor-associated antigens and OVA. However, more direct analyses of the impact of histamine receptors on B cell function have suggested that in the absence of both H1 and H2 function B cell activity is inhibited, at least in the context of IgE responses in allergic sensitization (27); this is in marked contrast to our findings of the impact of selective H2 blockade on IgG responses. The reported presence of H1 and H2 receptors on B cells provides opportunities for the modulation of a multitude of processes which may contribute to altered antibody responses, from B cell development and class switch through to antibody generation by plasma cells. The critical importance of B cells to the mechanism of action of ranitidine, demonstrated in this study, together with our previous evidence of MDSC involvement (10) could suggest distinct MDSC and B cell-dependent pathways, whereby ranitidine enhances effective antitumor immunity, or an important impact of MDSC on antitumor B cell responses. The latter would be more consistent with current literature on the direct impact of H2 receptors on B cell activities. In our study of B cell markers by flow cytometry, a decrease in B2 cell populations was noted in the inguinal draining lymph nodes of ranitidine-treated tumor-bearing mice compared with those left non-treated. As there was no significant change in the number of cells in the inguinal lymph node, this could signify that there is a skewing in the lymph node population toward other cell types, such as T cells. Furthermore, studies have shown that some B cells may be pro-tumorigenic, due to the release of immune suppressive cytokines such as IL-10 and TGFβ [reviewed in Ref. (28)] while others participate in antitumor responses. Therefore, there is potential that the decrease in B cells could be a decrease in certain subsets of B cells that are more pro-tumorigenic, leading to an increased proportion of antitumorigenic B cells.

A number of Fc receptor-bearing cells can contribute to antitumor immune effector processes in the presence of appropriate antibodies. There are some clinical reports which suggest that ranitidine may decrease neutrophil counts (29), particularly in children (30). Therefore, neutrophil and eosinophil numbers in the blood of ranitidine-treated and control tumor-bearing animals were tracked. No significant changes were observed as a result of ranitidine treatment, although the number of granulocytes was influenced by the tumor burden. NK cells also express Fc receptors and are well known to be able to kill tumor cells by mechanisms that are enhanced by the presence of antibodies to tumor-associated antigens (31). We decided to look at the splenic NK cells as a marker of a systemic effect ranitidine may have on NK cells, as well as the fact that we saw decreased monocytic MDSCs in the spleen (10), and MDSCs can have suppressive activity against NK cells (32, 33). Our findings of altered numbers of the intermediately functionally mature NK subsets might be indicative of greater NK cell turnover in ranitidine-treated mice. This may require further examination in future studies.

The effect of H2 antagonism on antibody levels may be due to direct or indirect effects on multiple stages of B cell function and antibody production. B cells express H2 receptors (20) and in the B cell-deficient mice, we did not see the inhibition of E0771-GFP tumor growth that we saw in the wild-type mice, suggesting that B cells are important for the inhibition of tumor growth. As our previous experiments in CCR2^−/−^ mice and in mice treated with gemcitabine (10, 11) show a lack of an enhanced antibody response with ranitidine treatment, it would suggest that in the E0771-GFP model, there is potential for indirect actions of ranitidine on B cell responses *via* MDSCs that would require further investigation. However, since ranitidine treatment can also enhance antibody responses to immunization in the absence of tumors as indicated by our OVA-alum immunization experiments. There may also be more direct impacts of ranitidine on B cells. T cells and a number of antigen-presenting cells also express histamine receptors so the precise mechanism of action of ranitidine in this context may be multifaceted. Notably, OVA in the context of B16 melanoma cells did not induce a significant IgG response, even in the context of ranitidine. This may relate to the lack of an adjuvant or a small effective antigen dose. It also remains possible that off-target effects of ranitidine treatment could contribute to altered antibody responses to tumors. However, we have previously demonstrated that oral treatment with alternate H2 antagonists, such as famotidine, inhibit breast tumor growth and metastasis in a manner similar to ranitidine (10).

Overall, this study illustrates the importance of histamine receptor antagonist treatment in modifying antitumor immunity and demonstrates the critical importance of B cell responses in mediating the impact of such treatment. This may be occurring both directly through histamine receptors on B cells and indirectly. Remarkably little information is available concerning the impact of such “over the counter” therapies on the long-term outcomes of breast cancer or the responses to immunotherapies. Our study underlines the importance of moving forward with examination of the role of H2 receptor blockade on tumor immunity in a clinical context.

## Ethics Statement

This study was carried out in accordance with the guidelines of the Canadian Council on Animal Care. All procedures were approved by the Dalhousie University Committee on Laboratory Animals.

## Author Contributions

DR performed the majority of experimental procedures, developed the concept of B cell dependency as well as completing examination of NK cell populations, evaluated and interpreted results, and wrote part of the manuscript. JM conceptualized and organized the planning and development of the study, supervised research activities and progress, evaluated and interpreted results, and wrote part of manuscript. A-VL developed ranitidine treatment model of inhibited tumor growth and antibody detection methodology, completed many of the initial studies, developed staining protocols and interpreted results, and wrote part of manuscript. AP completed the analysis of granulocyte populations in tumor bearing and control mice, interpreted results, and assisted with other experiments for this study. SO developed essential animal tumor models, assisted with development of flow cytometry methodology and interpretation of flow cytometry data, and interpreted results. PM developed spontaneous breast tumor model, performed experiments with this model and interpreted results. All authors reviewed and commented on the manuscript.

## Conflict of Interest Statement

The authors declare that the research was conducted in the absence of any commercial or financial relationships that could be construed as a potential conflict of interest. The reviewer LD and the handling Editor declared their shared affiliation.
