# Supplementary figures and images for: Ranitidine Inhibition of Breast Tumor Growth Is B Cell Dependent and Associated With an Enhanced Antitumor Antibody Response
Source: Front Immunol. 2018 Aug 15;9:1894. doi: 10.3389/fimmu.2018.01894 (PMC6104125; doi:10.3389/fimmu.2018.01894)

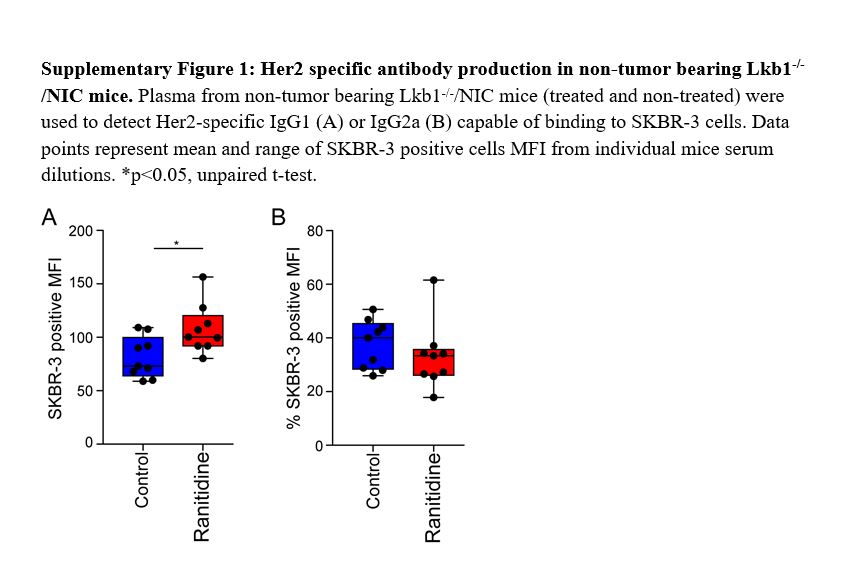

Supplement: Supplementary file 1 [file image_1.jpeg]
