# Supplementary material for: Ranitidine Inhibition of Breast Tumor Growth Is B Cell Dependent and Associated With an Enhanced Antitumor Antibody Response
Source: Front Immunol. 2018 Aug 15;9:1894. doi: 10.3389/fimmu.2018.01894 (PMC6104125; doi:10.3389/fimmu.2018.01894)
Supplement: Supplementary file 2 [file table_1.docx]

Supplementary Table 1: B cell populations in splenocytes for wild-type E0771-GFP tumor-bearing mice. N=4

| E0771-GFP | | | |
| --- | --- | --- | --- |
|  | | Wild-type | |
|  |  | Control | Ranitidine |
| % CD19^+^CD43^-^ of live | Mean | 33.16 | 33.03 |
|  | SEM | 3.26 | 4.45 |
| % CD19^+^CD43^+^ of live | Mean | 7.26 | 5.11 |
|  | SEM | 1.19 | 0.10 |
| % CD23^int^CD21^+^ of CD19^+^CD43^-^ | Mean | 6.19 | 9.29 |
|  | SEM | 0.87 | 1.14 |
| % CD23^+^CD21^-^ of CD19^+^CD43^-^ | Mean | 78.41 | 78.07 |
|  | SEM | 3.86 | 1.30 |
